# Soil degradation and prevention in greenhouse production

**DOI:** 10.1186/2193-1801-2-S1-S10

**Published:** 2013-12-11

**Authors:** Y Liang, X Lin, S Yamada, M Inoue, K Inosako

**Affiliations:** Institute of Soil & Water Conservation, Northwest A&F University, Yangling, Shaanxi China; Institute of Soil & Water Conservation, the Chinese Academy of Sciences & Ministry of Water Resources, Yangling, Shaanxi China; Faculty of Agriculture Tottori University, 4-101, Koyama-chominami, Tottori 680-8553 Japan; Arid Land Research Center, Tottori University, Hamasaka, Tottori, Japan

**Keywords:** Soil degradation, greenhouse production, cropping model, soil bio-characteristics, preventing from soil degradation

## Abstract

Soil degradation has been a very serious problem for sustainable production, especially by a re-cropping of greenhouse-cultivated cucumber (*Cucumis statirus* L.). The aim of this research was to expound the actuality for soil degradation, at the same time, put forward some suggestion for preventing from soil degradation and maintain sustainable production in greenhouse basic on the two experiments conducted in a solar greenhouse during 2001-2008 suburb area of Yan'an, Shaanxi province in North China. The result shown that cucumber fruit productivity increased as the increasing of re-cropping years, but decreased after 5years continuously cropping. As increasing of re-cropping years, the population of fungus and bacteria increased, which was assumingly main factor of soil degradation. There was significant difference in cropping models on soil bio-characteristics and system productivity. The productivity were the highest in cropping model between cucumber and greengrocery, cucumber and cowpea *(Vigna sinensis L)*, the second higher were in cropping model between cucumber and maize (*Zea mays*) for green manure, cucumber and kidney bean *(Phaseolus vulgaris)*. That was the best way to reduce soil bacteria and epiphyte amount to follow lasting three or four months during summer season after cucumber harvest, the better method was planting cowpea or other leguminous crops. Basic on the experiment, the optimums approaches to preventive soil degradation were put forward.

Solar greenhouse vegetable cultivation has been proven to be a good farming practice in various areas of different countries [1–3], and has been developed rapidly during recent years because of the comparatively higher economic benefit [4–7].

Soil degradation is the universal problem in greenhouse soil. It affects the sustainable use of greenhouse soil and also endangers vegetable security.

## Actuality of soil degradation in greenhouse

### Soil salinity

Greenhouse vegetable cultivation often requires a greater degree of management and larger input of nutrients and irrigation. However, over-fertilization and over-irrigation for vegetable cultivation resulted in nutrient accumulation in soil and led to soil salinity [8, 9]. Salinity reduces phosphate uptake and accumulation in crops grown in soils primarily by reducing phosphate availability. Salinity can directly affect nutrient uptake, such as Na^+^ reducing K^+^ uptake or by Cl^-^ reducing NO_3_^-^ uptake. Salinity can also cause a combination of complex interactions that affect plant metabolism, susceptibility to injury or internal nutrient requirement. Salinity has been described as the 'AIDS of the soil' and its influence is spreading throughout society where crop production has been seriously affected and caused economic hardship.

### Continuous cropping

As the development of specialized vegetable production, continuous cropping is inevitable in greenhouses. A lot of vegetable such as tomato (*Lycopersicum esculentum*), pepper (*Capsicum frutescens*), eggplants (*Solanum melongena*), and cucumber have frequently suffered continuous cropping obstacle [10]. At the same time, poorly differentiated plant mass getting into the soil under continuous cropping negatively affects soil organism communities [11]. This negative action leads to the change in metabolism of microbes from the primary to the secondary [12]. Continuous cropping obstacle has became a main problem for sustainable production of vegetable, for example, serious plant diseases and fall of yield, which intimidated facility vegetables production sustainable development, especially in North China [5, 13–17].

### Soil acidification

A number of agricultural practices have expanded the areas of acidification soils. The main causal factor is the growth of plants that use large amounts of basic ions (e.g. legumes); particularly when fertilizers that leaves acidic residues (such as Superphosphate) are used.

### Nutrition accumulation

At present, fertilizer application rates in intensive agricultural systems have increased dramatically in recent years, especially in greenhouse vegetable production systems [18]. These high fertilizer inputs and the extremely low crop recoveries of fertilizer nutrients lead to marked deterioration in soil and groundwater quality and the systems are clearly unsustainable [19]. Soil chemical and biological properties in greenhouse vegetable production may change dramatically after several years of continually high inputs of fertilizers and irrigation water and planting of shallow rooting vegetable crops compared with open field cereal production systems. Soil OM, alkalihydrolyzable N, and available P and K have been observed to accumulate significantly in vegetable greenhouses [5]. High concentrations of P and imbalances of N, P and K often occur in the intensively managed soils. High fertilizer application rates may lead to increasing salinity in the surface soils of the plastic film greenhouses in which vegetables are grown [20].

### Compaction

Some researcher found that bulk density was higher in greenhouse soils than uncovered soil. In recent years, there has been increasing interests in intensive vegetable cultivation, this caused the soil compacted. Compaction of soils causes a reduction in soil pore space. This reduces the rate at which water can infiltrate and drain through the soil. It also reduces the available space for oxygen in the plant root zones. For this reason, some of the major consequences of compaction are poor drainage, poor aeration, and hard pan surfaces which cause runoff. Repeated cultivation of some soils leads to a breakdown of soil structure and this also increases the likelihood of compaction.

### Chemical residue

Although not as large a problem as some of the other types of soil degradation, the presence of chemical residues can be quite a problem on a local scale. These residues derive almost entirely from long term accumulation after repeated use of pesticides, etc., or of use of pesticides or other chemicals with long residual effects. Some problems that result from chemical residues include toxic effects on crop species and contamination of workers, livestock and adjacent streams.

The studies on the soil degradation of greenhouse soil and its affection mechanism on vegetables are highly important for us to maintain high land capacity. For a sustainable management of soil in the greenhouse, it is urgent to improve the fertilizer and irrigation management for vegetable production, especially for the winter season under greenhouse conditions. Excessive nitrogen application can be effectively avoided by site-specific fertilizer application based on soil and plant analysis.

**Prevention from soil degradation in greenhouse**

## Materials and methods

The two experiment were conducted in a solar greenhouse during 2001-2008 suburb area of Yan'an, Shaanxi province in North China, a typical loess hill-gullied region of the Loess Plateau Longitude E109°19'23", Latitude N36°51'30", with an elevation of 1068-1309 m, mean temperature of 8.8°C, the annual precipitation of 500 mm. In the experiment 1, in 2001, the plots were filled with continuously cropped typical sandy soils of winter-spring season cucumber for 0year (hereafter referred to as yr), 1yr, 4yr, or 8yrs, respectively with 4 replications. The plots area was 15.6 m^2^. A grafted cucumber with the squash root was transplanted to the continuous cropped soil. The cucumber fruit productivity and species of soil microbes were analyzed. In the experiment 2, the 7 kinds cropping models between winter-spring season cucumber and summer other different crops on the productivity and soil bio-characteristics were studied. The cropping models were: cucumber and greengrocery, cucumber and cowpea, cucumber and maize for green manure, cucumber and kidney bean, cucumber and tomato (*Lycopersicum esculentum*), cucumber and dark bean (*Phaseolus*) for green manure, cucumber and fallow. The plots area was 39.6 m^2^ with three replications.

The soil moisture was maintained 75-90% as relative soil water capacity using drip irrigation system. The cucumber fruits were harvested sequentially from the early of December till the next year early of July. Microbe was determined by dilution-plate assay method, PDA broth medium used for soil fungus measured, beef extract peptone broth medium used for soil bacteria, starch-ammonium and martin broth medium used for soil actinamices [21]. To improve the precision of the results, every sample was measured three times, and the average value was calculated.

The experimental data was analyzed using SPSS software, tested with F-test means. Results were compared based on Duncan method (L.S.R).

## Results

### Cucumber fruit productivity in different re-cropping year

Table 1 showed that there was significant difference in cucumber productivity among re-cropping years, the treatment of 1yr, 2yr, 3yr and 4yr showed significant higher yield, the treatment after 5yr showed lower yield, and this difference had reached the statistics significant level (p = 0.01). It means that the productivity increased as re-cropping years increasing before re-cropping 4yr, but the yield decreased obviously after 5 yrs re-cropping, indicting that degradation of soil quality was occurred.

**Table 1:** The cucumber fruit yield in different re-cropping year (kg/m^2^)

Treat.	2002	2003	2004	2005	2006	2007	2008
0yr	12.76 B*	15.31 A	13.83 A	12.41 A	12.39 A	10.03 A	9.36 A
1yr	15.32 A	15.74 A	13.69 A	12.05 A	11.36 A	9.12 B	8.62 AB
4yr	13.95 B	11.23 B	9.65 B	8.32 B	8.16 B	8.28 C	8.23 B
8yr	11.86 C	10.23 B	9.31 B	8.36 B	8.21 B	8.13 C	7.86 B

* Notes: the letters in the same column show significant difference at 0.01 levels.

### Soil microbe in different re-cropping year

In the soil, fungus and bacteria increased greatly, but actinamices did not show a big change as re-cropping years increasing (Table 2), which led to a rise of occurrence rate of pathogenic microbe and soil-borne, and lead to a decrease of fruit productivity. Additionally, the change in microbe group affected nutrient conversion in the soil, which possibly became an obstacle for nutrient absorption by crops.

**Table 2:** Soil fungus, bacteria and actinamices content in different re-cropping year

Treat.	Bacteria 10^6^.g^-1^ds	Significant	Fungus 10^2^.g^-1^ds	Significant	Actinamices 10^4^.g^-1^ds	Significant
0yr	1.57	D*	3.8	D	20.75	A
1yr	1.48	D	1.2	E	11.13	B
3yr	1.03	E	5.4	C	10.49	B
5yr	2.95	C	4.2	D	8.19	C
7yr	3.32	B	6.3	B	3.53	D
9yr	8.90	A	7.2	A	3.43	D

* Notes: the letters in the same column show significant difference at 0.01 levels.

There are some means of soil quality rehabilitation. Reasonable rotation is the best way, for example, use of rotation system of cucumber with maize or dark-bean as green manure, rotation system of cucumber with cowpea. These crops can absorb different nutrients from soil, and abate the occurrence of diseases infected by soil through changing stubble [22]. Maintenance of adequate balance of fertilizers, addition of organic manure, and soil sterilization are secondary recommended for soil quality rehabilitation.

### Cucumber fruit productivity in different cropping models

The significant difference in cucumber fruit productivity among cropping models was showed in Table 3, during 2002-2008, the cropping models of cucumber with greengrocery, cucumber with cowpea showed significant higher yield, the second higher yield appeared in the cropping models between cucumber and maize for green manure, cucumber and tomato, cucumber and kidney bean, the cropping models of cucumber with dark-bean for green manure, cucumber with fallow showed lower yield, the difference had reached the significant level (p = 0.01).

**Table 3:** The cucumber fruit yield in different cropping models (kg/m^2^)

Treat.	2002-2003	2003-2004	2004-2005	2005-2006	2006-2007	2007-2008
Kidney bean	16.24 B*	16.17 AB	13.34 BC	17.30 B	16.70 B	16.40 C
Tomato	15.20 B	15.06 B	14.30 B	17.80 B	15.30 BC	15.10 CD
Fallow	13.40 C	11.91 C	11.63 C	13.90 C	12.50 C	14.80 D
Maize for green manure	16.23 B	13.27 BC	13.84 B	17.60 B	17.80 B	18.50 B
Dark-bean for green manure	14.02 C	10.92 C	12.62 C	15.30 C	14.10 C	16.60 C
Cowpea	17.91 A	17.26 A	14.97 B	18.60 B	20.80 AB	18.60 B
Greengrocery	20.05 A	19.24 A	20.44 A	21.50 A	22.30 A	20.80 A

* Notes: the letters in the same column show significant difference at 0.01 levels.

### Soil microbe in different cropping models

In the soil, the number of fungus and bacteria changed greatly when planting different crops during summer season after winter-spring season cucumber harvested (Table 4). The number of bacteria in the soil was the highest after planting tomato during summer season, the second higher number were in the soil after planting greengrocery, maize for green manure and dark-bean for green manure during summer season, the lowest number of bacteria were in the soil after planting kidney bean, cowpea and fallow. The number of fungus in the soil was higher after planting maize for green manure, dark-bean for green manure and kidney bean, the second higher were in the soil after planting tomato and greengrocery, the lowest number were in the soil after planting cowpea and fallow during summer season.

**Table 4:** Soil fungus and bacteria in different cropping models (18 Oct. 2002)

Treat	Bacteria 10^6^.g^-1^ds	5% Significant	Fungus 10^2^.g^-1^ds	1% Significant
Maize for green manure	56.94	b	88.97	A
Tomato	101.36	a	46.97	B
Cowpea	46.43	c	35.71	C
Kidney bean	43.59	c	95.89	A
Greengrocery	62.79	b	40.70	B
Dark-bean for green manure	48.84	bc	79.70	A
Fallow	41.21	c	25.45	C

These results indicated that it was effective method to use different cropping models for decreasing number of microbe in soil, to fallow three or four month or to plant leguminous rotation system during summer season.

## Conclusions

The productivity increased as re-cropping years increasing, but the yield decreased obviously after 5 yrs re-cropping, this indicted that degradation of soil quality was occurred. The increase of fungus and bacteria number became main factors of soil degradation as re-cropping years increasing. The cropping models of cucumber and greengrocery, cucumber and cowpea showed significant higher cucumber yield, the second higher yield appeared in the cropping models between cucumber and maize for green manure, cucumber and tomato, cucumber and kidney bean. Therefore, that was effective method to use different cropping rotation models for decreasing number of microbe in soil, to prevent from soil degradation in greenhouse to fallow three or four month or to plant leguminous during summer season after winter-spring season cucumber harvested.

## References

[1] Li W, Luo HY, Liu JF (1996). The material and energy fluxes in greenhouse ecosystem and the evaluation of its benefits. Ecology Agric Res (in Chinese).

[2] Nakano A, Yamauchi A, Uehara Y (2003). Effects of application of low-sulfate slow-release fertilizer (LSR) on shoot and root growth and fruit yield of tomato. Jpn Agric Res Qua.

[3] Darwish T, Atallah T, Moujabber ME, Khatib N (2005). Salinity evolution and crop response to secondary soil salinity in two agro-climatic zones in Lebanon. Agric Water Manage.

[4] Liao YC, Wang LX (1999). Facility Agriculture and the Building of Chinese Agricultural Modernization. Research of Agricultural Modernization (Chinese).

[5] Guo LS, Yang WC (2000). The understanding and fulfillment of Facility Agriculture. Sciences Research Management (Chinese).

[6] Feng GH (2000). Discussion on Facility Agriculture. Facility Agriculture (Chinese).

[7] Liang YL, Chen ZJ, Wang ZM (2002). The status and function of Facility Agriculture on Ecological Environment constructing. Journal of Soil and Water Conservation.

[8] Stigter TY, Ooijen SPJ, Post VEA, Applello CAJ, Dill AMMC (1998). A hydro geological and hydro chemical explanation under irrigated land in a Mediterranean environment. Algarve, Portugal J Hydrol (Amst).

[9] Chen Q, Zhang XS, Zhang HY, Christie P, Li XL, Horlacher D (2004). Evaluation of current fertilizer practice andsoil fertilizer in vegetable production in the Beijin gregion. Nutr Cycl Agroecosyst.

[10] Takahashi K (1984). Injury by continuous cropping in vegetables: various problems in the cultivation using grafted plants. Yasaishikenjo Kenkyu Shiryo.

[11] Lech Szajdak (1996). Impact of crop rotation and phonological periods on rhodanese activity and free sulfuric amino acids concentrations in soils under continuous rye cropping and crop rotation. Environment International.

[12] Bu'Lock JD (1980). Mycotoxins as secondary metabolism. In: Steyn, P.S.,ed. Biosynthesis of mycotoxins.

[13] Liu D, Wu FZ (1998). Effects of the re-cropping on the roots activity and the photosynthesis rate of the plastic greenhouse cucumbers. Journal of Northeast Agricultural University.

[14] Liang YL, Chen ZJ, Xu FL (2003). The effect of re-cropping year on physiological characteristics of cucumber in sunlight greenhouse. Acta botanical boreali-occidentalia sinica.

[15] Liang YL, Chen ZJ, Xu FL, Zhang CE, Du SN, Yan YG (2004). Soil Re-cropping Obstacles in Facility Agriculture on Loess Plateau. Journal of Soil and Water Conservation.

[16] Yu JQ, Du YS (2000). The Re-cropping Obstacles Problems among The Sustainable Development of Vegetable Facility Gardening. Journal of Shenyang Agriculture University (Chinese).

[17] Wu FZ, Zhao FY, Liu YY, Li
 X.L., Zhang
 F.S., Mi
 G.H (2000). Analysis of the problems in continues cropping system of protective vegetable and the controlling ways. Fertilizing for Sustainable Production of High Quality Vegetables.

[18] Ju XT, Wan YJ, Kou CL, Zhang FS, Li (2005). Nitrate accumulation in agricultural soils and groundwater of the North China Plain. Plant Nutrition for Food Security, Human Health and Environmental Protection.

[19] Zhu JH, Li XL, Christie P, Li JL (2005). Environmental implications of low nitrogen use efficiency in excessively fertilized hot pepper (Capsicum frutescent L.) Cropping systems. Agriculture. Ecosystems and Environment.

[20] Wu FZ (2000). On the reasons of re-cropping obstacles in vegetable facility gardening. Journal of Northeast Agriculture.

[21] Zhou DQ (1983). Microbiology experiment Booklet. Shanghai, Science and technology press of Shanghai.

[22] Liang YL, You HX, Chen ZJ, Gao JY, Du SN, Zhou MJ, Chen JR, Xiong YM (2006). System productivity and soil bio-characteristics of different cropping model in Facility Agriculture. Journal of Soil and Water Conservation.

